# Selective Neurectomy of the Facial Nerve with Cross-Face Nerve Graft for Treating Postparalytic Facial Nerve Syndrome

**DOI:** 10.1055/a-2531-3083

**Published:** 2025-05-15

**Authors:** Ko Nakao, Takako Fujii, Hisashi Sakuma

**Affiliations:** 1 Department of Plastic and Reconstructive Surgery, Ichikawa General Hospital, Tokyo Dental College, Ichikawa, Japan

**Keywords:** facial nerve, contralateral facial nerve, facial palsy, neural signal augmentation

## Abstract

Although postparalytic facial nerve syndrome (PFS) is a frequent sequela of partial facial palsy, no effective treatment is currently available. Herein, we report a case of a cross-face nerve graft (CFNG) technique with selective neurectomy of the facial nerve in a 52-year-old female with moderate PFS (especially oral-ocular synkinesis and facial contracture) and a House–Brackmann score grade III. Selective neurectomy resulted in the release of the synkinesis and contractures. Furthermore, we reinnervated the levator muscles of the upper lip and oral commissure by connecting the contralateral facial nerve to the thick zygomatic branch of the facial nerve via a CFNG, which allowed neural signal augmentation of the levator muscles. No obvious PFS recurrence was observed 1 year postoperatively. This procedure is expected to provide a new treatment option for improving PFS because it is effective and less invasive.

## Introduction

Postparalytic facial nerve syndrome (PFS) is a sequela of peripheral facial nerve palsy that results in functional and cosmetic defects and often contributes to psychological stress. 1 The facial movement disorders occur due to significant facial nerve degeneration followed by aberrant or faulty nerve regeneration. Among the symptoms of PFS, oral-ocular synkinesis and facial contracture are particularly intractable, and botulism toxin injections and physical therapy have become the gold standard treatment. 2 Botulinum toxin injections are noninvasive but reversible and must be continued throughout life, and rehabilitation therapy does not provide significant improvement for the symptoms. 2

Recent reports have focused on treating PFS by selective neurectomies, and there have been several reports of good results obtained by neurectomy of the nerve branches, which are thought to be confused or overexcited. 3 4 However, selective neurectomy at the distal branches was thought to trigger PFS, and these prior reports did not discuss the possibility of restraining the remaining nerves in the long term. In addition, the associated theories predict a weakening of oral commissure excursion with neurectomy.

Herein, we report a case of a patient with PFS treated by simultaneous selective neurectomy of the zygomatic and buccal branches of the facial nerve that contracted the entire mimetic muscles. Among the transected nerves, the thick zygomatic branch running to the zygomaticus major muscle and the contralateral facial nerve were connected via a cross-face nerve graft (CFNG). We considered that selective neurectomy with the CFNG technique could simultaneously treat PFS and augment the levator function of the upper lip and oral commissure.

## Idea

**Video 1** Preoperative video. The symptoms of PFS, such as synkinesis and facial contractures, are readily apparent. PFS, postparalytic facial nerve syndrome.


**Video 2** One-month postoperative video. The symptoms of PFS showed improvement, although there was a concurrent weakening of the levator function of the upper lip. PFS, postparalytic facial nerve syndrome.


**Video 3** One-year postoperative video. The levator function has improved slightly due to neural signal augmentation using CFNG. CFNG, cross-face nerve graft.


A 52-year-old female developed right facial nerve palsy secondary to Bell's palsy. The patient had complete paralysis, so she was given oral steroids for 10 days and antiviral medication for 5 days from the onset. Although rehabilitation using biofeedback and facial muscle stretching recovered steadily, she developed PFS 6 months after the onset and visited our hospital. She had a deepening of the nasolabial fold at rest due to contracture, oral-ocular synkinesis, and mimetic stiffness on the paralyzed side ( Video 1 ). Since the symptoms were moderate, with a House–Brackmann score of grade III, selective neurectomy with CFNG neural signal augmentation was performed 1 year after onset.

The procedure was performed on an outpatient basis under general anesthesia. A standard rhytidectomy incision was made, and lidocaine hydrochloride was not administered to avoid inadvertent facial nerve paresis. The facial flap was elevated through a preauricular face-lift incision in the plane of the superficial musculoaponeurotic system. The facial nerve branches were identified at the anterior margin of the parotid gland, and each branch was traced to the periphery to identify the muscle innervation area and communication branches. Using facial nerve monitoring electrodes (Medtronic, Goleta, CA) in combination with a nerve stimulator (Keisei Medical Industrial Co., Tokyo), each branch was stimulated (0.5–2.0 mA) to confirm the extent of mimetic muscle contraction.

The facial neuroanatomy procedure described by Freilinger et al 5 indicated that there are usually two zygomatic branches. The thin cranial branch (Z1) has few subbranches and independently controls the middle of the orbicularis oculi muscle, and the thick caudal branch (Z2) runs medially under the zygomaticus major muscle while also having a large communicating branch (ZB) with buccal branches (B). Z2 mainly controls the zygomaticus major but also partially runs to the orbicularis oculi muscle. 6

**Fig. 1:**
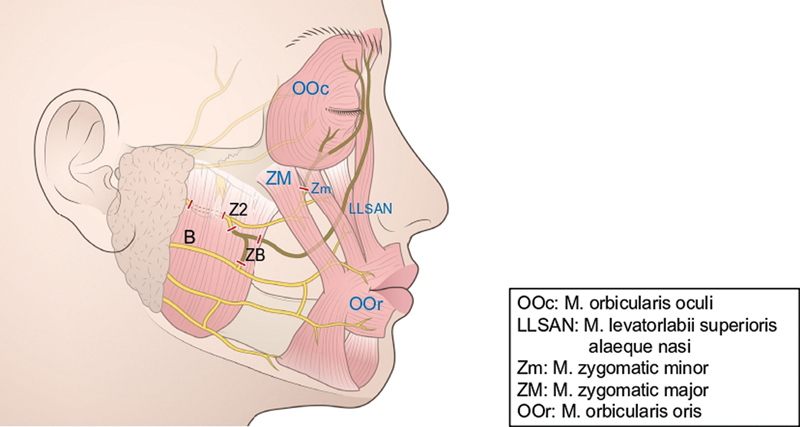
Operative schema (1). Z2 and ZB, including the traffic branches, which cause simultaneous mimetic muscle contraction, were resected. The branch of Z2 toward the orbicularis oculi muscle was also transected. Z2, thick caudal branch; ZB, large communicating branch.

Z2 and ZB, which cause the mimetic muscle to contract strongly and simultaneously upon electric stimulation, were resected, and the branch of Z2 toward the orbicularis oculi muscle was also transected. The Z2 was transected at the anterior border of the parotid gland for neural transfer. The eyelid closure function was retained by preserving Z1, which independently contracts the orbicularis oculi muscle, and the lip-closing function was retained by preserving ZB ( Fig. 1 ).

**Fig. 2:**
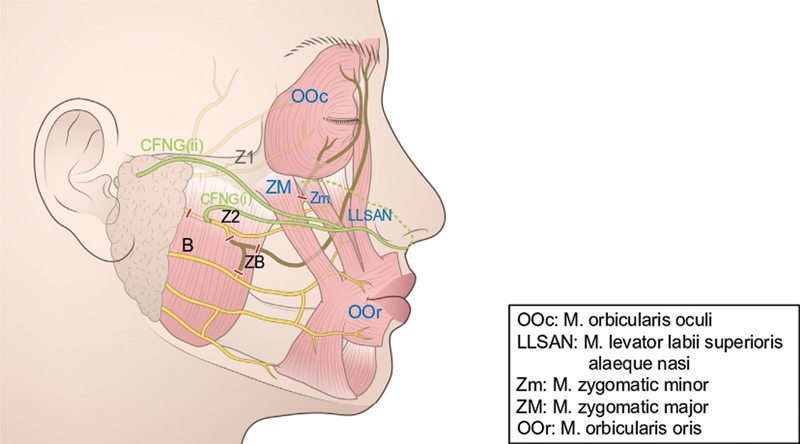
Operative schema (2). Z2 was transacted at the anterior border of the parotid gland and connected to the contralateral zygomatic branch via CFNG to reinnervate the zygomaticus major/minor and levator labii/anguli. CFNG, cross-face nerve graft.

**Fig. 3:**
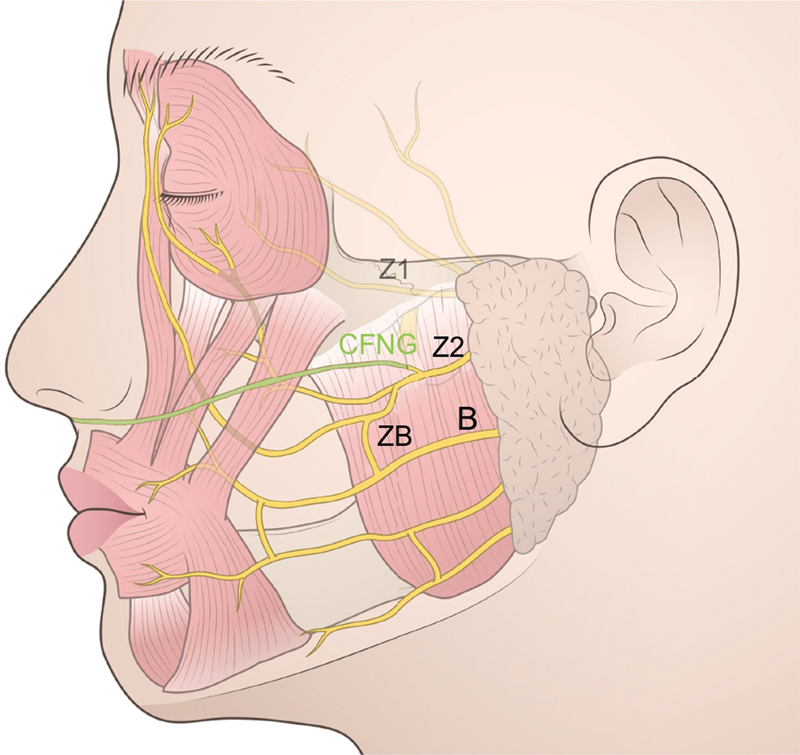
Operative schema (3). The contralateral zygomatic branch uses one of the branches of Z2.

**Fig. 4:**
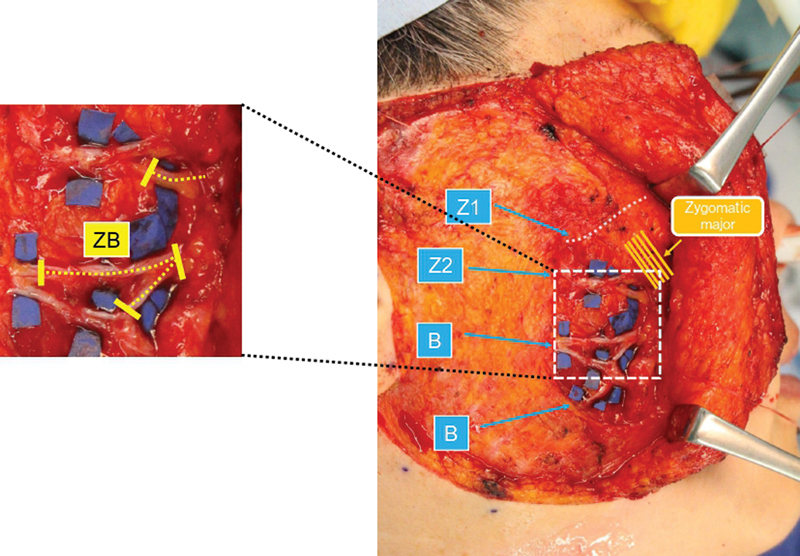
Selective neurectomy. Neurectomy of the ZB, which contracted the mimetic muscle. ZB is a large communicating branch.

**Fig. 5:**
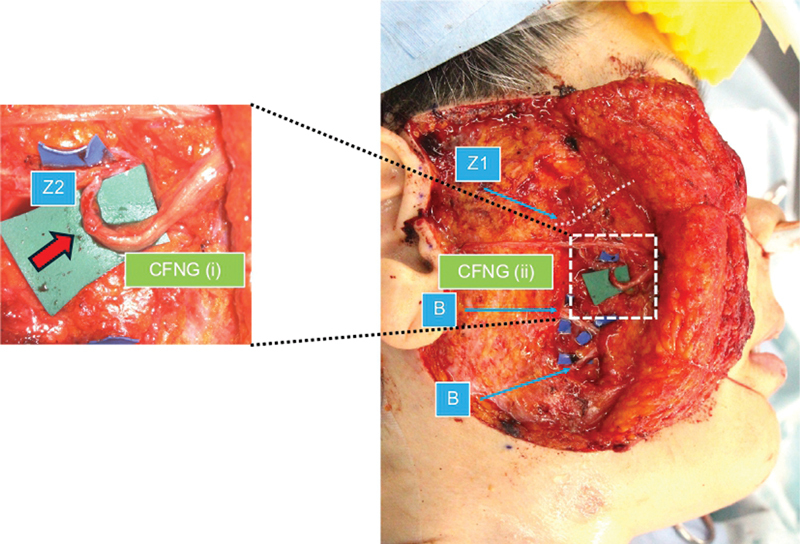
Neural signal augmentation with CFNG. The CFNG was split in two on the affected side of the face into CFNG (i) and CNFG (ii). The transected Z2 was sutured end-to-end to the CFNG (i) at the subzygomatic triangle (red arrow). CFNG (ii) was fixed subcutaneously in the preauricular region. Z2, thick caudal branch. CFNG, cross-face nerve graft.

The sural nerve was taken from the foot through a series of small transverse incisions of about 15 to 17 cm. The levator muscles of the upper lip and oral commissure, such as the zygomaticus major/minor and levator labii/anguli, were reinnervated by suturing to the Z2 and contralateral zygomatic branch via a sural nerve ( Fig. 2 ). The contralateral zygomatic branch is the branch that corresponds to Z2 in anatomy. If it is directly connected to Z2, the affected side will become paralyzed, so one of the several branches of Z2 is used ( Fig. 3 ). The sural nerve was split in two using CFNG on the affected facial region, one (CFNG [i]) was sutured to the transected Z2 and the other (CFNG [ii]) was subcutaneously placed in the preauricular region ( Fig. 2 ). CFNG (ii) was prepared subcutaneously to be used as a motor nerve for an additional free muscle transfer, if the smile movement was poor postoperatively, although this turned out not to be necessary. Intraoperative findings are shown in Figs. 4 and 5 .

**Fig. 6:**
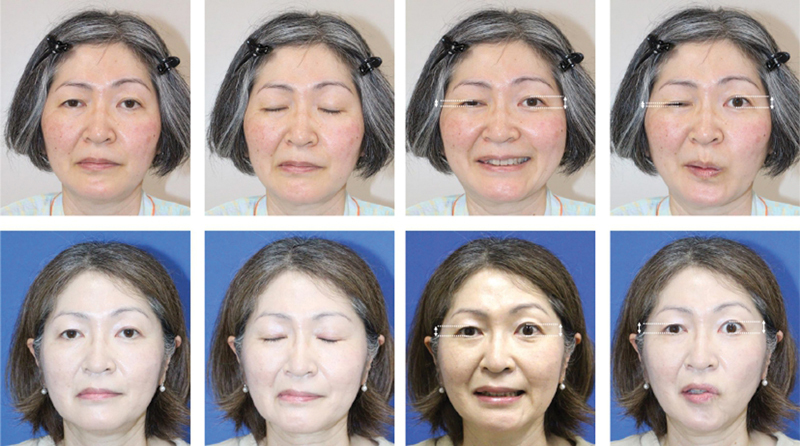
Pre- and 1-year postoperative evaluations. Upper row: preoperative images, lower row: 1-year postoperative images. The deepening of the nasolabial fold due to the contracture had improved and became symmetrical. No weakening of the eye or mouth closing function was observed. The ratio of the palpebral fissure height (affected/normal) improved from 0.46 to 0.82 in smile and from 0.25 to 0.75 in lip pucker.

The patient was evaluated by two board-certified plastic surgeons 1 year postoperatively. The eyelid closure and mouth closing functions remained almost the same as before surgery. Due to the effect of the neurectomy, the movement of the orbicularis oris has been slightly weakened, making it hard to make a strong o-shape, but the patient does not feel any particular inconvenience in their daily life, such as eating or talking. The deepening of the nasolabial fold due to facial contracture also improved and became symmetrical ( Fig. 6 ). The ratio of the palpebral fissure height (PFH) on the normal and affected sides was measured preoperatively and postoperatively to assess the treatment efficacy on oral-ocular synkinesis. The PFH ratio (affected/normal) improved from 0.46 (preoperatively) to 0.82 (postoperatively) in an open smile and from 0.25 (preoperatively) to 0.75 (postoperatively) in lip pucker.

**Fig. 7:**
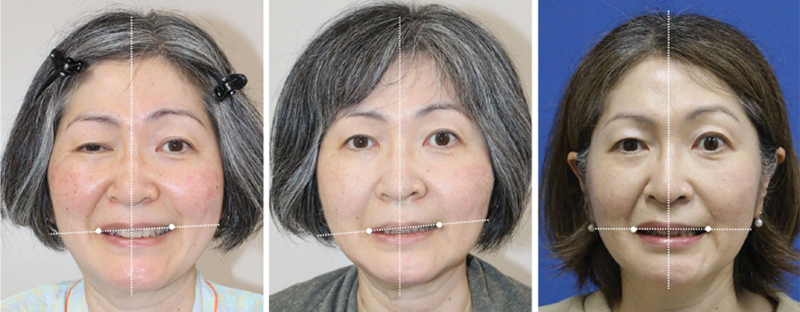
Pre- and postoperative evaluations. Left: preoperative image, center: 1-month postoperative image, right: 1-year postoperative image. CFNG prevented the weakening of the upper lip levator function during neurectomy. The height of the angle of the mouth remained the same or improved slightly.

Fig. 7 shows the levator function of the upper lip before surgery, 1 month after surgery, and 1 year after surgery. The corners of the mouth, which temporarily drooped after the neurectomy ( Video 2 ), showed improvement 1 year after the surgery due to the effect of neural signal augmentation from the contralateral facial nerve ( Video 3 ). The Sunnybrook facial grading system score improved from 58 (voluntary movement 76, resting symmetry 10, synkinesis 2) to 79 (voluntary movement 80, resting symmetry 0, synkinesis 1). The mimetic stiffness symptoms also decreased, and the patient was satisfied with the results.

Written informed consent was obtained from the patient for publication of this case report and accompanying images and video.

## Discussion

The concept of facial nerve neurectomy was first reported for hemifacial spasm by Coleman et al in 1937 7 ; however, its application to facial nerve palsy sequelae was first reported by Marino et al in 1950. 8 Hohman et al 3 described selective neurectomy for orbicularis oculi synkinesis. Azizzadeh et al 4 reported that the natural smile mechanism was reconstructed by selective neurectomy of the buccal branch innervating the depressor anguli oris (DAO), which is an antagonistic muscle of the smile. In this study, we preserved the nerves that innervate and caused relatively poor contraction of the orbicularis oculi and orbicularis oris, and the thick zygomatic and buccal branches, which have anatomically developed networks, were centrally resected. 9 Chen et al 10 reported that in rats, facial nerves are already zone-labeled on the trunk and run with a slight overlap. If the neural network gradually decreases toward the periphery and independently innervates the muscles, it seems reasonable to perform a neurectomy in the central region, which is expected to have the most crosstalk.

The main cause of facial contracture is not over-tension of the muscle itself but over-reinervation with synkinesis; therefore, central neurectomy is appropriate for reducing axonal input to the entire facial muscle. In cadavers, nerve branches to the orbicularis oculi that are divided during selective neurectomy can be redirected to target the zygomaticus major muscle to reinforce the smile. 11 Based on this report, we considered a method to power the zygomaticus major muscle by connecting the transected nerve to the contralateral facial nerve and simultaneously filling the space at the nerve cut end so that the regenerative nerve would not stray.

The CFNG is often compared with the hypoglossal or masseter nerve as the donor nerve for the facial nerve. The hypoglossal nerve has an excellent resting tone 12 ; however, in cases where the patient originally had a strong contracture, there is concern about contracture recurrence and synkinesis due to new input from the hypoglossal nerve. While the CFNG creates a smile in synchronization with the contralateral side, the hypoglossal nerve creates a smile upon tongue movement, which is unnatural and likely to cause discomfort because tongue movement is constant and often involuntary.

Spira et al 13 first introduced the use of the masseter nerve in facial reanimation through free neuromuscular transfer in 1978, and this technique has recently become popular. Coombs et al 14 reported that the masseter nerve contained 1,542.67 axons on average in a series of seven patients undergoing facial nerve palsy surgery. The volume of this nerve is reported to be twice that of the buccal branch and 15 times that of the CFNG. Therefore, while the masseter nerve can provide powerful and reliable motor signals for facial reanimation, there are also reports that the motion is rather too strong and causes discomfort. 15 16 Furthermore, there is concern that the resting tone cannot be maintained with masseter innervation alone, and involuntary oral commissure movements during meals can continue to persist. 17

In contrast, CFNG is useful for creating a smile spontaneously and can also create certain resting tones. 15 However, there is concern that the distal stump of the CFNG has fewer nerve fibers than the masseter nerve, thus resulting in weak movement of the smile. Therefore, we divided the CFNG into two parts: one part was sutured end-to-end to the Z2 area, and the other was subcutaneously implanted in the anterior ear region, enabling the CFNG to function as a motor nerve in the event that a free muscle transfer was needed in the future. In our case, the patient did not require a free muscle transfer using the CFNG, as no significant left–right difference was noted in the postoperative smile.

It is necessary to take 15 to 17 cm in length of the sural nerve as an interposition nerve, but the postoperative sequelae are limited to partial sensory paresthesia of the external condyle, so the sacrifice is considered to be minimally significant.

Recently, some studies have reported on treatments that improve the function of smiling by selective myectomy of the DAO and mentalis, which are considered to be the antagonistic muscles of smiling. Nevertheless, this technique is deemed efficacious solely in instances where the hypertonicity of antagonistic muscles is pronounced. 18 19 In contrast, our method can be applied to all cases without exception.

Notably, this is the first report of selective neurectomy with neural signal augmentation for the treatment of PFS. This technique should be considered an alternative to less invasive treatment options for patients who develop synkinesis with partial facial palsy.

This study has some limitations that should be noted when interpreting our findings. First, this approach is not sufficient for severe paralyzes with House–Brackmann scores of grade IV or higher, which require reconstruction with free muscle transfer in addition to selective neurectomy. Second, the patient was followed up for 1 year postoperatively. The possibility of recurrence of PFS due to nerve regeneration from the remaining peripheral branches cannot be ruled out, and long-term follow-up is necessary.

To conclude, in this study, we considered selective neurectomy of the network between the thick zygomatic and buccal branches, which causes synkinesis and contracture, and connected the thick zygomatic branch to the contralateral facial nerve in a patient with moderate PFS. Selective neurectomy ensured symmetry at rest, and CFNG input simultaneously prevented the weakening of the levator function. The CFNG technique also prevented the recurrence of PFS by straying pathologically regenerating axons into the cut end. This is a useful procedure that can be added to the armamentarium for facial reanimation.
